# Luminescence of *Cypridina* Luciferin in the Presence of Human Plasma Alpha 1-Acid Glycoprotein

**DOI:** 10.3390/ijms21207516

**Published:** 2020-10-12

**Authors:** Shusei Kanie, Mami Komatsu, Yasuo Mitani

**Affiliations:** Bioproduction Research Institute, National Institute of Advanced Industrial Science and Technology (AIST), Hokkaido Center, Sapporo 062-8517, Japan; komatsu.2525@aist.go.jp

**Keywords:** *Cypridina* luciferin, cypridinid luciferin, human plasma alpha 1-aicd glycoprotein, *Cypridina* luciferase, cypridinid luciferase, bioluminescence

## Abstract

The enzyme *Cypridina* luciferase (CLase) enables *Cypridina* luciferin to emit light efficiently through an oxidation reaction. The catalytic mechanism on the substrate of CLase has been studied, but the details remain to be clarified. Here, we examined the luminescence of *Cypridina* luciferin in the presence of several proteins with drug-binding ability. Luminescence measurements showed that the mixture of human plasma alpha 1-acid glycoprotein (hAGP) and *Cypridina* luciferin produced light. The total value of the luminescence intensity over 60 s was over 12.6-fold higher than those in the presence of ovalbumin, human serum albumin, or bovine serum albumin. In the presence of heat-treated hAGP, the luminescence intensity of *Cypridina* luciferin was lower than in the presence of intact hAGP. Chlorpromazine, which binds to hAGP, showed an inhibitory effect on the luminescence of *Cypridina* luciferin, both in the presence of hAGP and a recombinant CLase. Furthermore, BlastP analysis showed that hAGP had partial amino acid sequence similarity to known CLases in the region including amino acid residues involved in the drug-binding ability of hAGP. These findings indicate enzymological similarity between hAGP and CLase and provide insights into both the enzymological understanding of CLase and development of a luminescence detection method for hAGP.

## 1. Introduction

**Figure 1:**
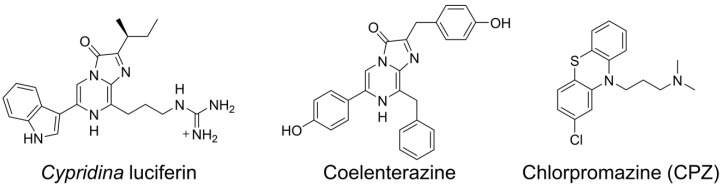
Chemical structures of compounds used in this study.

The luminous ostracods of the family Cypridinidae, commonly called sea fireflies, produce blue light (λmax = 448–463 nm depending on the buffer composition) by an enzyme-catalyzed chemical reaction with excellent quantum yield (Φ_BL_ = ~0.30) [1,2,3]. In the bioluminescence reaction, *Cypridina* luciferin (Figure 1), recently called cypridinid luciferin, is oxidized in the presence of *Cypridina* luciferase (CLase), recently called cypridinid luciferase, and molecular oxygen (oxidation step), followed by generation of the oxyluciferin in the excited state (excitation step) and subsequent change to the ground state with light emission (light production step) [3,4,5]. The molecular mechanism of *Cypridina* bioluminescence has been studied [3] since Hirata’s group determined the structure of *Cypridina* luciferin after many great efforts to study *Cypridina* bioluminescence in the first half of the nineteenth century [6,7,8]. For understanding the mechanism in terms of the enzyme, CLase genes were cloned from *Vargula* (*Cypridina*) *hilgendorfii* and *Cypridina noctiluca*, and these recombinant CLases were characterized [9,10]. Recently, transcriptome analyses of cypridinid ostracods that inhabit the Caribbean Sea or the coast of California identified thirteen putative CLases [11], and an efficient expression system of a recombinant CLase was developed [12]. However, the protein crystal structure of CLase has yet to be solved, and the catalytic mechanisms, including knowledge of the active sites, remain to be clarified. To date, some applications of *Cypridina* bioluminescence have been reported [13], but the poor enzymological understanding of the bioluminescence is one obstacle hindering future applications.

To understand how the reaction of *Cypridina* luciferin with CLase results in efficient luminescence, researchers have focused on the chemiluminescence features of *Cypridina* luciferin or its analogs under various chemical conditions [14,15,16,17,18,19,20]. In 1966, Johnson et al. reported spontaneous luminescence of *Cypridina* luciferin in dimethyl sulfoxide (DMSO) without CLase [21] while such luminescence was slightly observed in an aqueous solution in the absence of CLase [22]. After these findings, Goto et al. reported that *Cypridina* luciferin or its analogs emitted light more efficiently in diethylene glycol dimethyl ether containing acetate buffer (pH 5.6) or Tris buffer (pH 9.0) containing cetyltrimethylammonium bromide than in DMSO [23,24]. In addition, fluorometric titration analyses of the oxidized *Cypridina* luciferin–CLase complex indicated that *Cypridina* luciferin was surrounded by a hydrophobic environment in CLase [25]. From these observations, Goto et al. hypothesized that, for *Cypridina* bioluminescence, CLase plays an important role not only in promoting the reaction of *Cypridina* luciferin with molecular oxygen but also in providing *Cypridina* luciferin with a hydrophobic environment to emit light efficiently [24,26].

Supporting this hypothesis, previous papers have reported luminescence of *Cypridina* luciferin or its imidazopyrazinone-type analogs in the presence of molecules that have hydrophobic cavities responsible for their compound-binding abilities (e.g., cyclodextrins or serum albumins) [15,27,28,29,30,31,32,33]. The mechanism of luminescence in the presence of a serum albumin or a cyclodextrin is not well understood, but it is suggested that the hydrophobic cavities of these molecules are involved [15,30]. For further understanding of *Cypridina* bioluminescence through Goto’s hypothesis, we focused on human plasma alpha 1-acid glycoprotein (hAGP), also called orosomucoid. The glycoprotein hAGP is a plasma protein-like serum albumin and has one hydrophobic cavity responsible for its binding to basic drugs [34,35,36]. Chlorpromazine (CPZ) is one of the basic drugs that bind to hAGP (Figure 1), and the cocrystal structure analysis showed that CPZ bound to the hydrophobic cavity [35]. Given the basic guanidine moiety found in *Cypridina* luciferin and that the luminescence of *Cypridina* luciferin analogs depended on fitting to cyclodextrin’s hydrophobic cavity [15,27], we conjectured that *Cypridina* luciferin would bind to hAGP with high affinity and would emit light more efficiently than in the presence of a serum albumin.

In this study, we examined the luminescence of *Cypridina* luciferin in the presence of hAGP. We showed that the luminescence intensity of *Cypridina* luciferin in the presence of hAGP was higher than in the presence of ovalbumin (OVA), human serum albumin (HSA), or bovine serum albumin (BSA). In addition, we found that hAGP had partial amino acid sequence similarity to CLases from *C. noctiluca* and *V. hilgendorfii* in the region including amino acid residues responsible for the drug-binding ability of hAGP.

## 2. Results

### 2.1. Luminescence of Cypridina Luciferin in the Presence of hAGP

**Figure 2:**
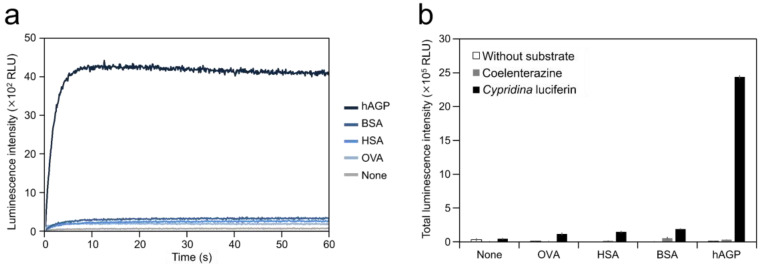
Luminescence of *Cypridina* luciferin in the presence of human plasma alpha 1-acid glycoprotein (hAGP): (**a**) luminescence kinetics of *Cypridina* luciferin in the presence of various proteins and (**b**) luminescence intensity of *Cypridina* luciferin or coelenterazine in the presence of various proteins. None, in the absence of any proteins; OVA, ovalbumin; HSA, human serum albumin; BSA, bovine serum albumin; hAGP, human plasma alpha 1-acid glycoprotein; and CLase, a recombinant *Cypridina* luciferase from *C. noctiluca*. The bars represent the mean values ± SD for *n* = 3.

To test the ability of hAGP to cause luminescence in *Cypridina* luciferin, we measured luminescence from a mixture of *Cypridina* luciferin with hAGP. Luminescence measurement showed that the mixture of *Cypridina* luciferin and hAGP in Tris-HCl (pH 7.5) exhibited glow-type light emission (Figure 2a). The total value of the luminescence intensity over 60 s was 19.9, 16.1, and 12.6-fold higher than those for the mixtures of *Cypridina* luciferin with OVA, HSA, and BSA (Figure 2b and Table 1). In contrast, no luminescence was observed from the mixture of coelenterazine, another imidazopyrazinone-type luciferin (Figure 1), with hAGP in Tris-HCl (pH 7.5) (Figure 2b). A comparison of hAGP and CLase showed that the total value of luminescence intensity of *Cypridina* luciferin over 60 s in the presence of 2.5 μg (approximately 106 pmol) of hAGP was 122-fold lower than that in the presence of 0.25 ng (approximately 4 fmol) of a recombinant CLase from *C. noctiluca* (Table 1).

### 2.2. Luminescence Property of Cypridina Luciferin in the Presence of hAGP

**Figure 3:**
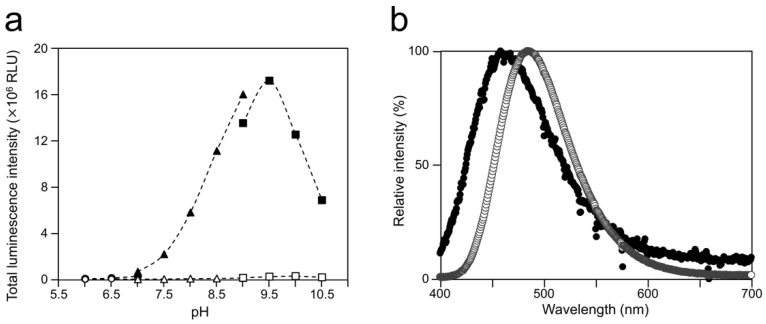
Optimum pH for luminescence of *Cypridina* luciferin with hAGP and spectra at the optimum pH: (**a**) luminescence of *Cypridina* luciferin in the presence of hAGP under various pH buffer conditions and (**b**) emission spectra in the presence of hAGP or a recombinant CLase from *C. noctiluca* in 50 mM Gly-NaOH (pH 9.5). In panel (**a**), closed circles represent 50 mM BisTris-HCl containing hAGP, open circles represent 50 mM BisTris-HCl, closed triangles represent 50 mM Tris-HCl containing hAGP, open triangles represent 50 mM Tris-HCl, closed squares represent 50 mM Gly-NaOH containing hAGP, and open squares represent 50 mM Gly-NaOH. The bars represent the mean values ± SD for *n* = 3. In panel (**b**), closed circles represent the presence of hAGP and open circles represent the presence of CLase.

**Figure 4:**
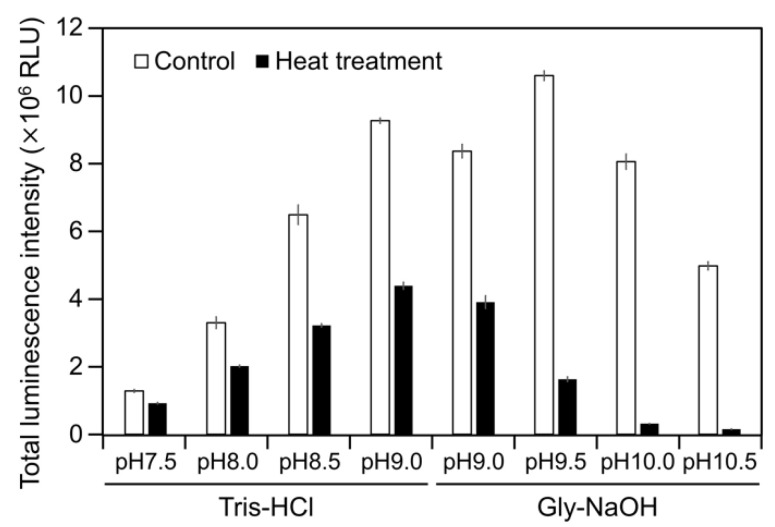
Luminescence intensity of *Cypridina* luciferin in the presence of intact or heat-treated hAGP under various pH buffer conditions. Control, without heat treatment; heat treatment, heated at 95 °C for 30 min. The bars represent the mean values ± SD for *n* = 3.

**Figure A1:**
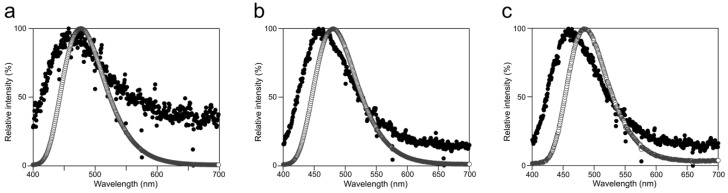
Luminescence emission spectra of *Cypridina* luciferin in the presence of hAGP or a recombinant CLase from *C. noctiluca*: spectra (**a**) in 50 mM Tris-HCl (pH 7.5), (**b**) in 50 mM Tris-HCl (pH 8.5), and (**c**) in 50 mM Gly-NaOH (pH 10.5). Closed circles, with hAGP; open circles, with CLase.

**Figure A2:**
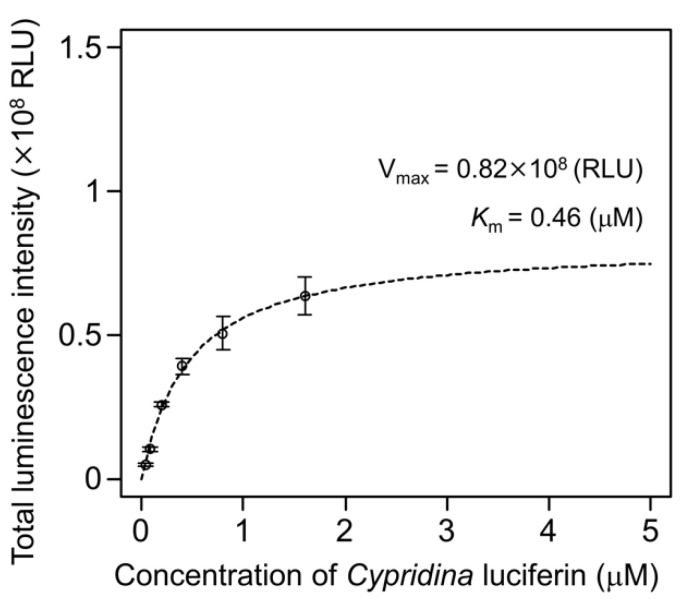
Luminescence intensity of various concentrations of *Cypridina* luciferin with hAGP under a Gly-NaOH (pH 9.5) buffer condition: the bars represent the mean values ± SD for *n* = 3.

**Figure A3:**
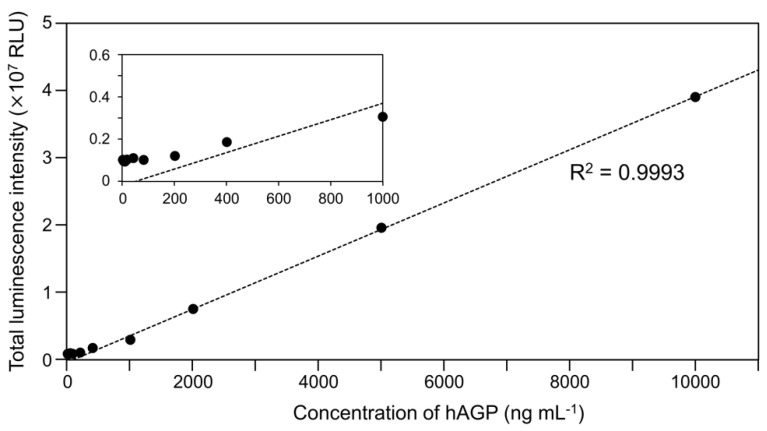
Luminescence intensity of *Cypridina* luciferin with various concentrations of hAGP under a Gly-NaOH (pH 9.5) buffer condition: quantitative linearity (correlation efficient R^2^ = 0.9993) was observed from 400 ng mL^−1^ to 10 mg mL^−1^. The bars represent the mean values ± SD for *n* = 3. The inset shows an enlarged view of the range from 0–1000 ng mL^−1^.

To examine the luminescence properties of the *Cypridina* luciferin-hAGP mixture, we measured luminescence intensity under various conditions. We observed luminescence in the range from pH 7.0 to 10.5 (Figure 3a). The maximum total value of the luminescence intensity over 60 s was obtained with Gly-NaOH (pH 9.5) buffer condition (Figure 3a). Under pH 7.5, 8.5, 9.5, and 10.5 buffer conditions, the maximum emission wavelength was 457 or 458 nm (Figure 3b, Figure A1 and Table A1). Although *Cypridina* luciferin showed chemiluminescence in the absence of hAGP at pH 9.0, 9.5, 10.0, and 10.5, the maximum total value of the luminescence intensity over 60 s was 60-fold lower than that in the presence of hAGP (Figure 3a). By heat treatment of hAGP at 95 °C for 30 min under pH 7.5–10.5 buffer conditions, the luminescence intensity of *Cypridina* luciferin decreased compared to that in the presence of intact hAGP (Figure 4). Under Gly-NaOH (pH 9.5) buffering conditions, as the concentration of *Cypridina* luciferin increased, luminescence intensity in the presence of hAGP increased and reached a plateau (Figure A2). Assuming that hAGP acted as a luciferase for *Cypridina* luciferin, the analysis of Michaelis–Menten kinetics of hAGP for *Cypridina* luciferin was performed and showed that the Km value was 0.46 μM (Figure A2). Luminescence measurements of the mixtures of *Cypridina* luciferin with various amounts of hAGP showed that the values of the total luminescence intensity over 60 s were proportional to the concentration of hAGP from 400 ng mL^−1^ to 10 μg mL^−1^ with a correlation efficient of 0.9993 (Figure A3).

### 2.3. CPZ Effect on Luminescence of Cypridina Luciferin in the Presence of hAGP

**Figure 5:**
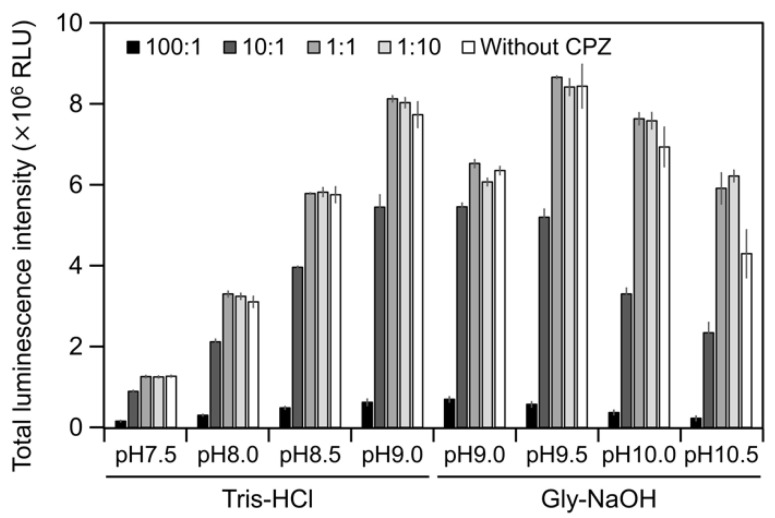
Luminescence intensity of *Cypridina* luciferin in the presence of hAGP with or without Chlorpromazine (CPZ) under various pH buffer conditions: the ratios indicate the molar ratio of CPZ to 6 pmol of *Cypridina* luciferin; without CPZ represents in the presence of only 6 pmol of *Cypridina* luciferin. The bars represent the mean values ± SD for *n* = 3.

**Figure A4:**
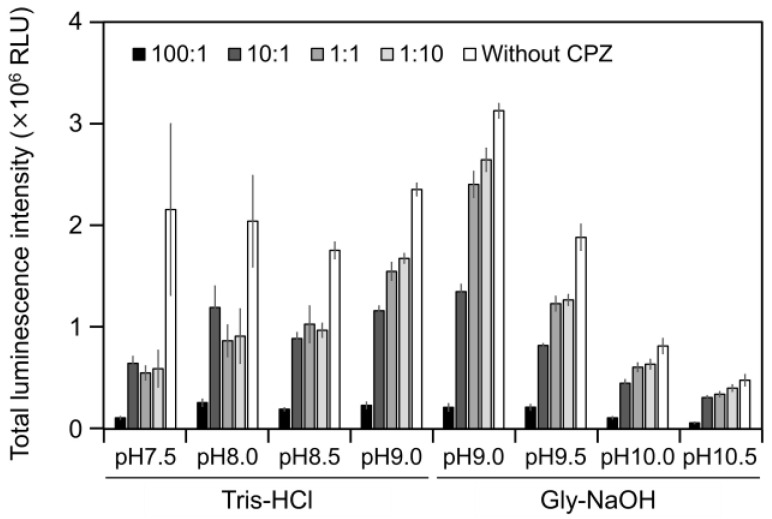
Luminescence intensity of *Cypridina* luciferin in the presence of a recombinant CLase from *C. noctiluca* with or without CPZ under various pH buffer conditions: the ratios indicate the molar ratio of CPZ to 6 pmol of *Cypridina* luciferin; without CPZ represents in the presence of only 6 pmol of *Cypridina* luciferin. The bars represent the mean values ± SD for *n* = 3.

**Figure A5:**
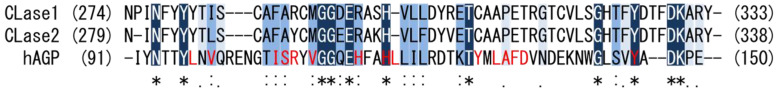
Partial alignment of hAGP with CLases in the region where the amino acid sequence similarity was found in BlastP analysis: CLase1 and CLase2 indicate CLase from *C. noctiluca* and CLase from *V. hilgendorfii*, respectively. Gaps are represented as dashes (-). Numbers in parentheses indicate the positions of initial and last amino acid residues. Red characters indicate amino acids involved in the drug-binding ability of hAGP [34]. Navy-shaded characters marked with an asterisk (*) indicate identical amino acid residues. Steel blue-shaded characters marked with a colon (:) indicate sites belonging to group exhibiting strong similarity. Light blue-shaded characters marked with a period (.) indicate sites belonging to a group exhibiting weak similarity. The site similarity followed the criteria of ClustalW [51].

To estimate the interaction of *Cypridina* luciferin with the drug-binding pocket in hAGP, we measured luminescence in the presence of hAGP with CPZ (Figure 1) under various pH conditions. When CPZ was present in the molar ratio of 100:1 or 10:1 (CPZ–*Cypridina* luciferin), the total values of the luminescence intensity over 60 s were lower than in the absence of CPZ under each buffer condition (Figure 5). Under the condition using a recombinant CLase from *C. noctiluca* instead of hAGP, the presence of CPZ in molar ratios of 100:1, 10:1, 1:1, or 1:10 (CPZ–*Cypridina* luciferin) also decreased the total values of luminescence intensity over 60 s compared to the absence of CPZ (Figure A4). BlastP analysis between hAGP (GenBank accession numer: CAA26397) and two CLases from *V. hilgendorfii* and *C. noctiluca* (AAA30332 and BAD08210) indicated that hAGP had a partial amino acid sequence similarity to these CLases in the region including amino acid residues involved in the drug-binding ability of hAGP, with E-values of 0.07 and 0.72, respectively (Figure A5 and Figure S1).

## 3. Discussion

In this study, we showed the luminescence of *Cypridina* luciferin in the presence of hAGP, a human plasma protein, under pH 7.5–10.5 buffer conditions (Figure 2 and Figure 3a). Luminescence intensity was highest at pH 9.5 (Figure 3a) and decreased in the presence of heat-treated hAGP compared to intact hAGP (Figure 4). These results did not reveal the role of hAGP in the luminescence process of *Cypridina* luciferin but suggested that hAGP exhibited enzymatic activity similar to that of CLase. When hAGP was considered an enzyme, its *K*m value for *Cypridina* luciferin was 0.46 μM, comparable to that of CLase (0.45 μM; Figure A2) [12], indicating that hAGP had a high affinity for *Cypridina* luciferin. This affinity probably enabled a low concentration of hAGP to interact with *Cypridina* luciferin to emit light (Figure 2 and Table 1). In contrast, insignificant luminescence was observed in the presence of the same concentrations of BSA or HSA (Figure 2 and Table 1), which were reported to cause luminescence of *Cypridina* luciferin analogs in previous studies [29,30,31,32]. This insignificant luminescence could be due to the lower affinity of both serum albumins to *Cypridina* luciferin, although efficiencies for each step of oxidation, excitation, and light production in the luminescence reaction of *Cypridina* luciferin in the presence of BSA, HSA, and hAGP must be clarified through an exact calculation of the quantum yield. Despite the high affinity of hAGP to *Cypridina* luciferin, the luminescence intensity in the presence of hAGP was much lower than in the presence of a recombinant CLase (Table 1). This result indicated that hAGP was not a specialized protein like luciferase for *Cypridina* luciferin, but the reason for the weaker luminescence is still unclear. 

The luminescence spectra obtained from *Cypridina* luciferin in the presence of hAGP under various pH buffer conditions showed that λmax was in the range of 457–458 nm (Figure 3b and Figure A1 and Table A1), while that in the presence of a recombinant CLase was in the range of 478–483 nm (Figure 3b and Figure A1 and Table A1). Considering a previous report that λmax in luminescence of *Cypridina* luciferin depended on the surrounding environment [17,22], the difference implied that the environment inside each protein was different. However, we assumed that the character of the binding site of *Cypridina* luciferin in hAGP was similar to that of the catalytic site in CLase. This is because one possible explanation for the inhibitory effect by CPZ (Figure 5 and Figure A4) is that both catalytic sites for *Cypridina* luciferin inside hAGP and CLase are suitable for binding CPZ and therefore blocking the binding of *Cypridina* luciferin. Alternatively, a direct interaction of CPZ with *Cypridina* luciferin or electron-transfer quenching of the oxyluciferin in the excited state could reduce luminescence. Although the inhibitory effect on hAGP was observed only under excess CPZ conditions (CPZ–*Cypridina* luciferin = 100:1 or 10:1) (Figure 5), we cannot rule out the possibility that the CPZ-binding pocket of hAGP interacted with *Cypridina* luciferin considering the reported association constant of CPZ to hAGP, 232 ± 57 μM [37]. To clarify the inhibitory mechanism by CPZ, further investigation of the inhibitory effect by a CPZ analog that does not bind to hAGP or by a hAGP-binding compound structurally dissimilar to CPZ is required. 

As shown in Figure A5 and Figure S1, we found that hAGP had partial amino acid sequence similarity to CLases from *C. noctiluca* and *V. hilgendorfii* mainly in the region that includes amino acid residues involved in the drug-binding ability of hAGP [34]. When we focused on CLase, we noticed that the partial amino acid sequences probably included amino acid residues responsible for the luciferase activity based on the previous study of truncated CLases [38,39]. Therefore, the alignment analysis of hAGP with CLase also allowed us to assume that the character of the drug-binding pocket of hAGP was similar to that of the catalytic pocket of CLase. It was noteworthy that the similarity between hAGP and CLases was found only when searching for amino acid sequence alignment between hAGP and CLase on a one-to-one basis, while an exhaustive homology search using CLase as a query did not reveal significant similarity between hAGP and CLase. This suggests that a one-to-one homology search is an important approach to find proteins with latent luciferase activity.

In future work based on our findings, we expect to develop a clinical examination method to quantify hAGP. In addition, we hope to conduct a study in terms of molecular evolution. Recently, hAGP has attracted attention as a clinical biomarker, and thus, a more practical method for detecting hAGP in a clinical setting will be required [36,40]. As shown in Figure A3, the luminescence intensity of *Cypridina* luciferin was proportional to the concentration of hAGP, at least in the range from 400 ng mL^−1^ to 10 μg mL^−1^, and to the quantitative linearity range reliable to detect the concentration of hAGP in a disease state [36]. Xu et al. reported that luminescence of a *Cypridina* luciferin analog in the presence of HSA can be used for clinical application to quantify HSA [31]. Therefore, luminescence detection of hAGP using *Cypridina* luciferin would seem to be another promising method, although further experiments using clinical specimens will be necessary to confirm this. When we considered hAGP as a luciferase to be used for a reporter assay or in vivo imaging, the luminescence intensity from the combination of hAGP with *Cypridina* luciferin was much lower than that from known luciferin-luciferase systems (Table 1). However, because molecular evolutionary studies have reported better bioluminescence systems using a non-natural luciferase or a synthetic luciferin analog [41,42,43], molecular engineering of hAGP or synthetic improvement of *Cypridina* luciferin could produce a practical *Cypridina* luciferin-based hAGP detection system to observe biological processes. Possibly, since known luciferases do not originate from mammalian proteins, the hAGP-based system could allow us to observe an intact biological event in mammalian systems. In addition, the study of proteins homologous to luciferases has contributed to understanding the origin of luciferases [32,44,45,46]. Although we do not consider hAGP to be directly related to the origin of CLase, our finding of the partial similarity between hAGP and CLase could provide insight into the substrate recognition sites related to its critical function and could pave a way to seek the origin of CLase.

## 4. Materials and Methods

### 4.1. Materials

The commercially available materials used in this study were obtained from the following commercial suppliers. Human alpha 1-acid glycoprotein (alpha 1-acid glycoprotein from human plasma; lot number: SLBJ6840V) was from Sigma-Aldrich (St. Louis, MO, USA). OVA (albumin from egg; lot number: CAL1231), BSA (albumin from bovine serum, fatty acid/IgG/protease free; lot number: CAQ4454), coelenterazine, chlorpromazine hydrochloride, Tris-HCl buffers, glycine, sodium hydroxide, and hydrochloric acid were from FUJIFILM Wako Pure Chemical Corporation (Osaka, Japan). HSA (albumin, human serum, F-V; lot number: M8H7013) was from Nacalai Tesque (Kyoto, Japan). *Cypridina* luciferin was from ATTO Corporation (Tokyo, Japan). BisTris was from Dojindo Laboratories. All materials were used without further purification. A recombinant CLase from *C. noctiluca* was prepared according to the method reported previously [12]. 

### 4.2. Measurement of Luminescence Intensity

The luminescence intensity of the reaction mixtures in white 96-well plates (Eppendorf microplate 96/F-PP; Eppendorf, Hamburg, Germany) was measured using a luminometer (Phelios AB-2350; ATTO, Tokyo, Japan) and recorded in relative light units (RLU) in 0.1-s intervals over 60 s at room temperature. 

### 4.3. Measurement of Luminescence Intensity of Cypridina Luciferin in the Presence of Proteins

To 50 μL of a 50 ug mL^−1^ solution of OVA, HSA, BSA, or hAGP in 100 mM Tris-HCl (pH 7.5) or 50 μL of a 5 ng mL^−1^ solution of a recombinant CLase in 100 mM Tris-HCl (pH 7.5) in 96-well plates was added 50 μL of a 0.2 μM aqueous solution of *Cypridina* luciferin or coelenterazine using the auto injector in the Phelios luminometer, followed by immediate measurement of luminescence intensity at room temperature. Each reaction was performed in triplicate. The aqueous solutions of *Cypridina* luciferin and coelenterazine were prepared by diluting concentrated solutions with distilled water. Concentrations of *Cypridina* luciferin or coelenterazine in the concentrated solutions were determined spectrophotometrically using the reported molar absorption coefficients [3,47]. Concentrations of OVA, HSA, BSA, hAGP, and CLase in the buffer solutions were determined using the corresponding molar extinction coefficients at 280 nm, calculated with a peptide property calculator [48]. These solutions were prepared immediately prior to their use in this experiment.

### 4.4. Measurement of Luminescence Intensity of Cypridina Luciferin with hAGP under Various pH Conditions

To 50 μL of a 50 μg mL^−1^ solution of hAGP in 100 mM BisTris-HCl (pH 6.0, 6.5, and 7.0), Tris-HCl (pH 7.0, 7.5, 8.0, 8.5, and 9.0), or Gly-NaOH (pH 9.0, 9.5, 10.0, and 10.5) in 96-well plates was added 50 μL of a 0.2 μM aqueous solution of *Cypridina* luciferin using the auto injector in the luminometer, followed by immediate measurement of luminescence intensity at room temperature. Each reaction was performed in triplicate. The aqueous solution of *Cypridina* luciferin and the buffer solutions of hAGP were prepared as described in Section 4.3. 

### 4.5. Measurement of Luminescence Emission Spectrum

The luminescence emission spectra of *Cypridina* luciferin in the presence of hAGP or CLase under various pH buffer conditions were measured using a high sensitivity charge coupled device (CCD) spectrophotometer, LumiFLspectrocapture (AB-1850C; ATTO), with the following settings: measurement mode, single; measurement time, 1 min for CLase or 5 min for hAGP; slit width, 0.5 mm; camera gain, high; diffraction grating, 150 lines/mm; and shutter for measurement, automatic. To 50 μL of a 50 ug mL^−1^ solution of hAGP or a 50 ng mL^−1^ solution of a recombinant CLase in 100 mM Tris-HCl (pH 7.5 or pH 8.5) or Gly-NaOH (pH 9.5 or pH 10.5) in a 0.2 mL micro-tube (0.2 mL thin-walled tube; Thermo Fisher Scientific, MA, USA) was added 50 μL of a 0.2 μM (for CLase) or a 2 μM (for hAGP) aqueous solution of *Cypridina* luciferin manually, followed by immediate measurement of luminescence emission spectrum at room temperature.

### 4.6. Measurement of Luminescence Intensity of Cypridina Luciferin with Heat-Treated hAGP

To 30 μL of a 50 ug mL^−1^ solution of intact or heat-treated hAGP in 100 mM Tris-HCl (pH 7.5, 8.0, 8.5, and 9.0) or Gly-NaOH (pH 9.0, 9.5, 10.0, and 10.5) in 96-well plates was added 30 μL of a 0.2 μM aqueous solution of *Cypridina* luciferin using the auto injector in the luminometer, followed by immediate measurement of luminescence intensity at room temperature. Each reaction was performed in triplicate. The aqueous solution of *Cypridina* luciferin and the buffer solutions of hAGP were prepared as described in Section 4.3. Heat denaturation of hAGP (heat-treated hAGP) was performed in 100 mM Tris-HCl (pH 7.5, 8.0, 8.5, and 9.0) or Gly-NaOH (pH 9.0, 9.5, 10.0, and 10.5) at 95 °C for 30 min using a Block Bath Shaker (MyBL-100CS; AS ONE Corporation, Osaka, Japan).

### 4.7. Measurement of Luminescence Intensity of Cypridina Luciferin and CPZ with hAGP or CLase

To 30 μL of a 50 ug mL^−1^ solution of hAGP or a 25 pg mL^−1^ solution of a recombinant CLase in 100 mM Tris-HCl (pH 7.5, 8.0, 8.5, and 9.0) or Gly-NaOH (pH 9.0, 9.5, 10.0, and 10.5) containing 20, 2, 0.2, or 0.02 μM CPZ or without CPZ in 96-well plates was added 30 μL of a 0.2 μM aqueous solution of *Cypridina* luciferin using the auto injector in the luminometer, followed by immediate measurement of luminescence intensity at room temperature. Each reaction was performed in triplicate. The aqueous solution of *Cypridina* luciferin and the buffer solutions of hAGP and CLase were prepared as described in Section 4.3. Before the measurement of luminescence intensity, the solutions containing CPZ were incubated for 10 min at room temperature. 

### 4.8. Linear Regression Analysis between Luminescence Intensity of Cypridina Luciferin and Amount of hAGP

To 50 μL of a 6.4, 16, 32, 80, 160, 400, 800, 2000, 4000, 10,000, or 20,000 ng mL^−1^ solution of hAGP in 100 mM Gly-NaOH (pH 9.5) in 96-well plates was added 50 μL of a 1.6 μM aqueous solution of *Cypridina* luciferin by the auto injector in the luminometer, followed by immediate measurement of luminescence intensity at room temperature. Each reaction was performed in triplicate. The aqueous solution of *Cypridina* luciferin and the buffer solution of hAGP were prepared as described in Section 4.3. The obtained data were subjected to linear regression analysis using Excel (2016, Microsoft, Redmond, WA, USA). 

### 4.9. Kinetic Analysis of hAGP with Cypridina Luciferin

To 50 μL of a 50 μg mL^−1^ solution of hAGP in 100 mM Gly-NaOH (pH 9.5) in 96-well plates was added 50 μL of a 0, 0.08, 0.16, 0.4, 0.8, 1.6, or 3.2 μM aqueous solution of *Cypridina* luciferin using the auto injector in the luminometer, followed by immediate measurement of luminescence intensity at room temperature. Each reaction was performed in triplicate. The aqueous solution of *Cypridina* luciferin and the buffer solutions of hAGP and CLase were prepared as described in Section 4.3. The obtained data were subjected to kinetic analysis using the R program [49] to fit the Michaelis–Menten equation. 

### 4.10. Comparison of Amino Acid Sequences between hAGP and CLases

Protein similarity between hAGP and CLases was estimated using the BlastP program (National Center for Biotechnology Information) [50]. Alignment of hAGP with CLases was performed using ClustalW (DNA Data Bank of Japan) [51]. These analyses used the information of hAGP (CAA26397) and CLases from *C. noctiluca* and *V. hilgendorfii* (BAD08210 and AAA30332). 

## 5. Conclusions

We showed for the first time that hAGP enabled *Cypridina* luciferin to emit light and that hAGP had a partial amino acid sequence similarity to CLase in the region including amino acid residues involved in the drug-binding pocket of hAGP. These findings could help to break the current impasse in both fundamental and applied research on the *Cypridina* bioluminescence system, although further enzymological understanding of not only CLase but also hAGP in the presence of *Cypridina* luciferin is required. 

## Figures and Tables

**Table 1:** Luminescence of *Cypridina* luciferin in the presence of various proteins.

Protein	Amount of Protein (μg)	Luminescence Intensity (RLU)			
		Maximum Intensity		Integration over 60 s	
None	0	192 ± 94	(0.04%) ^1^	48,651 ± 7566	(0.02%) ^1^
OVA	2.5	330 ± 79	(0.06%) ^1^	122,820 ± 13,239	(0.04%) ^1^
HSA	2.5	319 ± 12	(0.06%) ^1^	151,578 ± 8893	(0.05%) ^1^
BSA	2.5	403 ± 14	(0.08%) ^1^	193,741 ± 5228	(0.07%) ^1^
hAGP	2.5	4426 ± 23	(0.84%) ^1^	2,439,420 ± 20,104	(0.82%) ^1^
CLase	0.25 (ng)	524,803 ± 56,087	(100%) ^1^	297,210,024 ± 31,692,238	(100%) ^1^

None, in the absence of any proteins; OVA, ovalbumin; HSA, human serum albumin; BSA, bovine serum albumin; hAGP, human plasma alpha 1-acid glycoprotein; CLase, a recombinant *Cypridina* luciferase from *C. noctiluca*. Mean ± SD for *n* = 3. Maximum intensity indicates maximum light intensity over 60 s. ^1^ Luminescence intensity relative to that of CLase (100% as 0.25 ng of protein used).

## Supplementary Materials

Supplementary materials can be found at https://www.mdpi.com/1422-0067/21/20/7516/s1. Figure S1: Overall alignment of hAGP with Clases.

## Abbreviations

CLase	*Cypridina* luciferase
hAGP	Human alpha 1-acid glycoprotein
λmax	Maximum emission wavelength
Φ_BL_	Quantum yield of bioluminescence
DMSO	Dimethyl sulfoxide
OVA	Ovalbumin
BSA	Bovine serum albumin
HSA	Human serum albumin
Tris	Tris(hydroxymethyl)aminomethane
Gly	Glycine
CPZ	Chlorpromazine
BlastP	Protein-protein basic local alignment search tool
RLU	Relative light unit
E-value	Expect value
SD	Standard deviation
HCl	Hydrochloric acid
BisTris	Bis(2-hydroxyethyl)iminotris(hydroxymethyl)methane
NaOH	Sodium hydroxide
CCD	Charge coupled device

## Appendix A

**Table A1:** Maximum emission wavelength with hAGP or CLase under various pH buffer conditions.

Protein	Buffer Conditions	λmax (nm)
hAGP	Tris-HCl buffer (pH 7.5)	458
	Tris-HCl buffer (pH 8.5)	457
	Gly-NaOH buffer (pH 9.5)	458
	Gly-NaOH buffer (pH 10.5)	458
CLase	Tris-HCl buffer (pH 7.5)	478
	Tris-HCl buffer (pH 8.5)	480
	Gly-NaOH buffer (pH 9.5)	484
	Gly-NaOH buffer (pH 10.5)	483
