# Supplementary material for: Luminescence of Cypridina Luciferin in the Presence of Human Plasma Alpha 1-Acid Glycoprotein
Source: Int J Mol Sci. 2020 Oct 12;21(20):7516. doi: 10.3390/ijms21207516 (PMC7588914; doi:10.3390/ijms21207516)
Supplement: Supplementary file 1 [file ijms-21-07516-s001.pdf]

## Supplementary Material

# Luminescence of *Cypridina* Luciferin in the Presence of Human Plasma Alpha 1-Acid Glycoprotein

**Shusei Kanie *, Mami Komatsu and Yasuo Mitani ***

Bioproduction Research Institute, National Institute of Advanced Industrial Science and Technology (AIST), Hokkaido Center, Sapporo, 062-8517

* Correspondence: s-kanie@aist.go.jp (S.K.) and mitani-y.aist.go.jp (Y.M.); Tel.: +81-11-857-8410 (S.K.)

```
BAD08210.1_CLase1      MKTLILAVALVYCATVHCQD-CPYEPDPPN---TVPTSCEAKEGECIDSS 46
AAA30332.1_Clase2      MKIIILSVILAYCVTDNCQDACPVEAEPPSSTPTVPTSCEAKEGECIDTR 50
CAA26397.1_hAGP        --------------------------------------------------

BAD08210.1_CLase1      CGTCTRDILSDGLCENKPGKTCCRMCQYVIECRVEAAGWFRTFYGKRFQF 96
AAA30332.1_CLase2      CATCKRDILSDGLCENKPGKTCCRMCQYVIECRVEAAGYFRTFYGKRFNF 100
CAA26397.1_hAGP        --------------------------------------------------

BAD08210.1_CLase1      QEPGTYVLGQGTKGGDWKVSITLENLDGTKGAVLTKTRLEVAGDIIDIAQ 146
AAA30332.1_CLase2      QEPGKYVLARGTKGGDWSVTLTMENLDGQKGAVLTKTTLEVAGDVIDITQ 150
CAA26397.1_hAGP        --------------------------------------------------

BAD08210.1_CLase1      ATENPITVNGGADPIIANPYTIGEVTIAVVEMPGFNITVIEFFKLIVIDI 196
AAA30332.1_CLase2      ATADPITVNGGADPVIANPFTIGEVTIAVVEIPGFNITVIEFFKLIVIDI 200
CAA26397.1_hAGP        ------------------------------MALSWVLTVLSLLPLLEAQI 20
                                                       . : :**:. :: *:  :*

BAD08210.1_CLase1      LGGRSVRIAPDTANKGMISGLCGDLKMMEDTDFTSDPEQLAIQPKINQEF 246
AAA30332.1_CLase2      LGGRSVRIAPDTANKGLISGICGNLEMNDADDFTTDADQLAIQPNINKEF 250
CAA26397.1_hAGP        P--LCANLVPVPITNATLDQITG-KWFYIASAFR------------NEEY 55
                           ...:.* . .:. :. : *   :         *           *:*:

BAD08210.1_CLase1      DGCPLYGNPDDVAYCKGLLEPYKDSCRNPINFYYYTISCAFARCMGGDER 296
AAA30332.1_CLase2      DGCPFYGNPSDIEYCKGLMEPYRAVCRNNINFYYYTLSCAFAYCMGGEER 300
CAA26397.1_hAGP        N---------------------KSVQEIQATFFYFTP------------- 71
                       :                     :   .   .*:*:*

BAD08210.1_CLase1      ASHVLLDYRETCAAPETRGTCVLSGHTFYDTFDKARYQFQGPCKEILMAA 346
AAA30332.1_CLase2      AKHVLFDYVETCAAPETRGTCVLSGHTFYDTFDKARYQFQGPCKEILMAA 350
CAA26397.1_hAGP        -------------NKTEDTIFLR-------------EYQTRQDQCIYNT 94
                                     :*..* .*             ::*   .: :  :

BAD08210.1_CLase1      DCFWNTWDVKVSHRNVDSYTEVEKVRIRKQSTVVELIVDGKQILVGGEAV 396
AAA30332.1_CLase2      DCYWNTWDVKVSHRDVESYTEVEKVTIRKQSTVVDLIVDGKQVKVGGVDV 400
CAA26397.1_hAGP        TYLNVQRENGTISRYVGGQEHFAHLLILRDTKTYMLAFDVNDEKNWGLSV 144
                              :  .   * * .   .. :: * :::..  * .* ::    *  *

BAD08210.1_CLase1      SIPYSSQNTSIYWQDGDILTTAILPEALVVKFNFKQLLVVHIRDPFDGKT 446
AAA30332.1_CLase2      SIPYSSENTSIYWQDGDILTTAILPEALVVKFNFKQLLVVHIRDPFDGKT 450
CAA26397.1_hAGP        YADKPETTKEQLGEFYEALDCLRIPKSDVVYTDWKK-------------- 180
                           .. ...    :  : *    :*:: **  ::*:

BAD08210.1_CLase1      CGICGNYNQDFSDDSFDAEGACDLTPNPPGCTEEQKPEAERLCNSLFAGQ 496
AAA30332.1_CLase2      CGICGNYNQDSTDDFFDAEGACALTPNPPGCTEEQKPEAERLCNNLFD-- 498
CAA26397.1_hAGP        -DKCEPLEKQHEKERKQEEGES---------------------------- 201
                        . *   ::: .:  : **.

BAD08210.1_CLase1      SDLDQKCNVCHKPDRVERCMYEYCLRGQQGFCDHAWEFKKECYIKHGDTL 546
AAA30332.1_CLase2      SSIDEKCNVCYKPDRIARCMYEYCLRGQQGFCDHAWEFKKECYIKHGDTL 548
CAA26397.1_hAGP        --------------------------------------------------

BAD08210.1_CLase1      EVPDECK 553
AAA30332.1_CLase2      EVPPECQ 555
CAA26397.1_hAGP        -------
```

**Figure S1.** Overall alignment of hAGP with Clases. CLase1 and CLase2 indicate CLase from *C. noctiluca* and CLase from *V. hilgendorfii*, respectively. Gaps are represented as dashes (-). Numbers in parentheses indicate the positions of last amino acid residues. Gray-shaded characters indicate amino acids involved in the drug-binding ability of hAGP [34]. An asterisk (*) indicate identical amino acid residues. A colon (:) indicate sites belonging to group exhibiting strong similarity. A period (.) indicate sites belonging to a group exhibiting weak similarity. The site similarity followed the criteria of ClustalW [51].
